# Mitochondrial Mechanisms of LRRK2 G2019S Penetrance

**DOI:** 10.3389/fneur.2020.00881

**Published:** 2020-08-25

**Authors:** Sylvie Delcambre, Jenny Ghelfi, Nassima Ouzren, Léa Grandmougin, Catherine Delbrouck, Philip Seibler, Kobi Wasner, Jan O. Aasly, Christine Klein, Joanne Trinh, Sandro L. Pereira, Anne Grünewald

**Affiliations:** ^1^Luxembourg Centre for Systems Biomedicine, University of Luxembourg, Esch-sur-Alzette, Luxembourg; ^2^Department of Oncology, Luxembourg Institute of Health, Luxembourg, Luxembourg; ^3^Institute of Neurogenetics, University of Lübeck, Lübeck, Germany; ^4^Department of Neuromedicine and Movement Science, Department of Neurology, St. Olav's Hospital, Norwegian University of Science and Technology, Trondheim, Norway

**Keywords:** leucine-rich repeat kinase-2 (LRRK2), G2019S, Parkinson's disease, penetrance, mitochondria, mitochondrial DNA (mtDNA), fibroblasts

## Abstract

Several mutations in leucine-rich repeat kinase-2 (LRRK2) have been associated with Parkinson's disease (PD). The most common substitution, G2019S, interferes with LRRK2 kinase activity, which is regulated by autophosphorylation. Yet, the penetrance of this gain-of-function mutation is incomplete, and thus far, few factors have been correlated with disease status in carriers. This includes (i) LRRK2 autophosphorylation in urinary exosomes, (ii) serum levels of the antioxidant urate, and (iii) abundance of mitochondrial DNA (mtDNA) transcription-associated 7S DNA. In light of a mechanistic link between LRRK2 kinase activity and mtDNA lesion formation, we previously investigated mtDNA integrity in fibroblasts from manifesting (LRRK2+/PD+) and non-manifesting carriers (LRRK2+/PD−) of the G2019S mutation as well as from aged-matched controls. In our published study, mtDNA major arc deletions correlated with PD status, with manifesting carriers presenting the highest levels. In keeping with these findings, we now further explored mitochondrial features in fibroblasts derived from LRRK2+/PD+ (*n* = 10), LRRK2+/PD− (*n* = 21), and control (*n* = 10) individuals. In agreement with an accumulation of mtDNA major arc deletions, we also detected reduced NADH dehydrogenase activity in the LRRK2+/PD+ group. Moreover, in affected G2019S carriers, we observed elevated mitochondrial mass and mtDNA copy numbers as well as increased expression of the transcription factor *nuclear factor erythroid 2-related factor 2* (*Nrf2*), which regulates antioxidant signaling. Taken together, these results implicate mtDNA dyshomeostasis—possibly as a consequence of impaired mitophagy—in the penetrance of LRRK2-associated PD. Our findings are a step forward in the pursuit of unveiling markers that will allow monitoring of disease progression of LRRK2 mutation carriers.

## Introduction

Parkinson's disease (PD) is the second most common neurodegenerative disorder with a prevalence of 1% over the age of 60 years old (1). The majority of cases are sporadic, and only 5–10% suffer from a familial form (2). PD is characterized by a progressive loss of dopaminergic neurons within the *substantia nigra*. The ensuing lack of neurotransmitter dopamine in the basal ganglia results in motor symptoms such as tremors, rigidity, bradykinesia, and postural instability (2).

To date, at least 12 genes have been unequivocally associated with the development of familial PD (3), including the gene coding for leucine-rich repeat kinase 2 (LRRK2) (4). There are seven definitely pathogenic mutations in LRRK2 (5). The most frequent genetic cause of PD, the G2019S substitution, is found in 4–5% of familial cases and ~1% of sporadic cases in the general population. Among Ashkenazi Jews and North African Arab Berbers, the mutation explains 15–40% of PD cases, respectively (1). By contrast, in Europe, only 1–7% of PD patients harbor this nucleotide change (6), and it is even rarer in Asian populations (2). The LRRK2 G2019S mutation is inherited with reduced penetrance (7). Thereby, the risk to develop motor symptoms increases with age, ranging from 28% at 59 years to 74% at 79 years (8). However, the molecular determinants of LRRK2-G2019S penetrance are largely unknown.

LRRK2 is a large protein (268 kDa) composed of 51 exons. Its enzymatic core comprises two main structures: a GTPase and a serine-threonine kinase domain. The G2019S mutation is situated in the kinase domain and increases LRRK2 kinase activity, which in turn causes hyperphosphorylation of LRRK2 targets (6). The cellular function of LRRK2 has not been fully elucidated, but there is evidence for an involvement of the protein in endocytosis, retromer complex modulation, autophagy, and mitochondrial homeostasis (2). Specifically, mutations in LRRK2 have been shown to interfere with the removal of mitochondria from microtubules during the initiation phase of mitophagy (9). In addition, altered respiratory chain function coinciding with increased oxidative stress and morphological changes has been observed in cellular models of LRRK2-PD (10). In the presence of the G2019S mutation, mitochondrial DNA (mtDNA) lesions accumulate in patient-derived neurons (11)—a process that can be reversed by kinase inhibitor treatment (12).

To monitor the movement disorder not only at the clinical but also at the molecular level, there are increasing scientific efforts to identify biological markers of LRRK2-PD onset and progression. However, thus far, few candidates have been described that distinguish PD patients with LRRK2 mutations and such carriers who do not (yet) show the typical hallmarks of the motor disorder. First, the assessment of LRRK2 phosphorylation rates in urinary exosomes revealed higher levels in manifesting (LRRK2+/PD+) compared to non-manifesting (LRRK2+/PD−) individuals harboring the common G2019S mutation in LRRK2 (13). Second, a linkage analysis in Tunisian Arab-Berbers identified a polymorphism in dynamin 3 *(DNM3)* as a penetrance modifier (14). Third, serum levels of the antioxidant urate were shown to be reduced in LRRK2+/PD+ compared to LRRK2+/PD− individuals (15). Fourth, mtDNA transcription was altered in LRRK2+/PD+ cases (16). Lastly, our own research previously demonstrated an increase in the mitochondrial reactive oxygen species (ROS) scavenger superoxide dismutase (SOD)2 (17) and an accumulation of somatic mtDNA major arc deletions in fibroblasts from LRRK2+/PD+ compared to LRRK2+/PD− individuals (18).

To further elucidate the role of the mitochondria in defining the penetrance of *LRRK2*-associated PD, we built on our published research in fibroblasts from controls, LRRK2+/PD−, and LRRK2+/PD+ cases. Extending the mtDNA integrity and oxidative stress analyses, we now assessed mtDNA abundance as well as functional parameters such as transcriptional and respiratory chain complex activities.

## Materials and Methods

### Study Cohort

Study participants were recruited at movement disorder clinics in Lübeck (Germany) and Trondheim (Norway). All participants gave written informed consent, and the study was approved by the local ethics committees. Genetic testing was performed as previously described (19). Individuals with the G2019S mutation in LRRK2 were examined by movement disorder specialists for clinical signs of PD. Mutation carriers diagnosed with PD according to the MDS Clinical Diagnostic Criteria were included in the LRRK2+/PD+ group. By contrast, non-manifesting carriers who did not fulfill these criteria were classified as LRRK2+/PD−. Demographic data of the cohort are summarized in Table 1. All individuals were of Caucasian descent.

**Table 1:** Demographics of the study cohort.

	**Controls**	**LRRK2+/PD−**	**LRRK2+/PD+**
*N*	10	21	10
Number of men (%)	4 (40%)	7 (33.33%)	4 (40%)
Mean age (*SD*), years	60.8 (13.11)	58.52 (15.35)	66.00 (12.45)
Median age (IQR), years	65 (54.75–69.00)	55 (52.5–66.5)	66 (57.25–77.5)
Mean age at onset (*SD*), years	-	-	55.89 (9.87)
Median age at onset (IQR), years	-	-	57.00 (48.00–64.00)

*LRRK2, leucine-rich repeat kinase-2; PD, Parkinson's disease; LRRK2+/PD−, non-manifesting LRRK2 G2019S mutation carriers; LRRK2+/PD+, manifesting LRRK2 G2019S mutation carriers; IQR, interquartile range; SD, standard deviation*.

### Cell Culture

Dermal fibroblasts from 10 LRRK2+/PD+ cases, 21 LRRK2+/PD− individuals, and 10 age-matched healthy controls were phenotyped. Fibroblasts were cultivated in Dulbecco's modified Eagle's medium (DMEM) high glucose without pyruvate (Life Technologies, 41965-039), supplemented with 12% fetal bovine serum (Life Technologies, 10500064) and 1% penicillin/streptomycin (Life Technologies, 15140163), and were incubated at 37°C and 5% CO_2_. Cells were split with Trypsin-EDTA (Life Technologies, 25300-096) when sub-confluent.

### Mitochondrial DNA Copy Number and 7S DNA Analysis

DNA was extracted using the QIAmp DNA Mini Kit (Qiagen, 51306) following the manufacturer's instruction. Transcription-associated 7S DNA and copy number were assessed using a real-time PCR approach based on TaqMan probes. A probe targeting the mtDNA gene mitochondrial NADH-dehydrogenase 1 (*MT-ND1*), located in the minor arc and typically spared from deletions, was measured relative to the nuclear-encoded single-copy gene beta-2-microglobulin (*B2M*) to quantify the amount of wild-type mtDNA copies (*MT-ND1:B2M*). In addition, with a probe targeting the non-coding region (NCR) of the mitochondrial genome, the proportion of transcriptionally active mtDNA molecules was assessed. During transcription, 7S DNA is incorporated in the NCR forming a triple-stranded displacement loop (D-loop) (20). By measuring the NCR relative to *MT-ND1*, the abundance of 7S DNA per mitochondrial genome can be determined.

Quantification was achieved using a dilution series of an internal standard. Multiplex real-time PCR was performed using genomic DNA, LightCycler 480 Probes Master reaction mix (Roche, 04707494001), TaqMan probes, and primers (Supplementary Table 1) as specified in the manufacturer's guidelines. The PCR reaction was run on a LightCycler 480 (Roche, 05015243001). The samples were denatured for 10 min at 95°C. Amplification ran over 45 cycles with a denaturation step of 10 s at 95°C, primer annealing of 30 s at 60°C, and elongation of 3 s at 72°C.

### Nuclear Factor Erythroid 2-Related Factor 2 and Mitochondrial DNA Gene Expression

RNA was extracted using the RNeasy Mini Kit (Qiagen, 74106) following the manufacturer's instructions. cDNA was synthesized using the SuperScript^TM^ III Reverse Transcriptase (Invitrogen, 18080044) using 400 ng of RNA as starting material. PCR was performed using iQ SYBR Green (Biorad, 170-8885). Primer sequences are shown in Supplementary Table 2. The expression of NADH dehydrogenase 1 (*MT-ND1*), NADH dehydrogenase 4 (*MT-ND4*), cytochrome b (*MT-CYTB*), cytochrome c oxidase (*MT-CO1*), and nuclear factor erythroid 2-related factor 2 (*Nrf2)* was normalized to β-actin (*ACTB*) expression. The PCR reaction was run on a LightCycler 480. The samples were denatured for 5 min at 95°C. Amplification ran over 45 cycles with a denaturation step of 10 s at 95°C, primer annealing of 10 s at 60°C, and elongation of 10 s at 75°C.

### Mitochondrial Function Assessment

#### Mitochondrial Isolation

Mitochondria were isolated from frozen fibroblast pellets from three LRRK2+/PD+ (mean age ± SD: 59.7 ± 5.7 years) and three LRRK2+/PD− (mean age ± SD: 65.3 ± 20.6 years) individuals. Briefly, pellets were washed and mechanically lysed with a pestle in homogenization buffer [10 mM Tris pH 7.4 (T1503, Sigma)], 1 mM ethylenediaminetetraacetic acid (EDTA, Sigma, E5134), and 250 mM sucrose (Sigma, 84100) with protease and phosphatase inhibitors (Thermo Fisher Scientific, 78440). Pure mitochondrial pellets were obtained after serial centrifugation steps and used in subsequent enzymatic assays.

#### Mitochondrial Enzyme Kinetics

NADH:ubiquinone oxidoreductase and cytochrome c oxidase activities were evaluated adapting well-established protocols, which measure the kinetics of NADH to NAD^+^ oxidation by complex I (21) or the oxidation of reduced cytochrome c by complex IV (22), respectively. We downsized the assays to 96-well plate format and used a microplate reader (BioTek Cytation 5) to follow absorbance. The detection of respiratory chain enzyme activities requires exposure of those mitochondrial enzymes, which was achieved by three cycles of snap freezing (liquid nitrogen) and thawing of the samples. Finally, complex I and IV activities were normalized to mitochondrial mass, which was determined by citrate synthase kinetic analysis as previously reported (23). Three (citrate synthase and complex IV) to six (complex I) independent replicates per sample were performed.

### Statistics

For statistical analyses, GraphPad Prism software (version 8.3.0) was used. The ROUT test was used to evaluate the presence of outliers. Datasets were then independently tested for the assumptions of parametric data. More precisely, normality (Shapiro–Wilk, D'Agostino, and Pearson tests) and homoscedasticity (Brown–Forsythe test) were evaluated. As parametric assumptions were not met, Mann–Whitney and Kruskal–Wallis (followed by Dunn's *post-hoc* test) tests were therefore utilized. Differences were considered significant (^*^) when *p*-values were below 0.05. Moreover, to assess the impact of age on the different outcomes, we estimated regression models with age as a covariate. We also tested the interaction of each outcome and age. However, these analyses indicated no impact or interaction of age on the reported results.

## Results

### Decreased Complex I Activity in Manifesting Carriers of the G2019S Mutation

**Figure 1:**
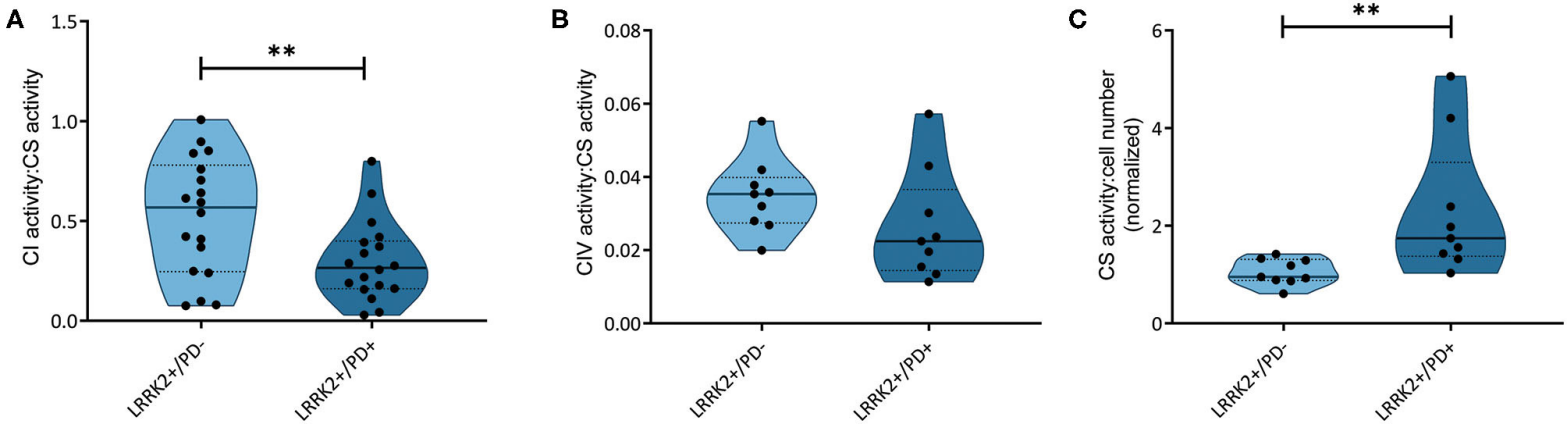
Respiratory chain complex and citrate synthase activities. **(A)** NADH dehydrogenase activity (complex I, CI; Mann–Whitney test: *p* = 0.003) and **(B)** cytochrome c oxidase (complex IV, CIV; Mann–Whitney test: *p* = 0.114) normalized to citrate synthase (CS) activity in non-manifesting [leucine-rich repeat kinase-2 (LRRK2)+/PD−, *n* = 3] and manifesting carriers (LRRK2+/PD+, *n* = 3) of the G2019S mutation in LRRK2. **(C)** CS activity relative to cell number, with one sample serving as an internal standard (Mann–Whitney test: *p* = 0.001). Experiments performed on six (CI) or three (CIV and CS) independent replicates. **p < 0.01.

We have recently reported that the accumulation of mtDNA deletions serves as a discriminator between affected and unaffected LRRK2 mutation carriers (18). The mtDNA deletions studied encompass *MT-ND4*, which codes for a subunit of complex I. To understand if those mtDNA deletions have an impact on respiratory chain function in LRRK2+/PD+ patients, we assessed the activity of complexes I and IV in a subset of the previously investigated samples. The quantification of NADH:ubiquinone oxidoreductase activity relative to citrate synthase activity showed significantly reduced complex I function (Mann–Whitney test: *p* = 0.003) in the LRRK2+/PD+ group [median: 0.26, interquartile range (IQR): 0.16–0.37] compared to the LRRK2+/PD− group (median: 0.49, IQR: 0.26–0.70) (Figure 1A). By contrast, analyzing cytochrome c oxidase activity relative to citrate synthase activity did not reveal differences between the two groups (LRRK2+/PD−, median: 0.035, IQR: 0.027–0.040; LRRK2+/PD+, median: 0.023, IQR: 0.014–0.037; Mann–Whitney test: *p* = 0.114) (Figure 1B).

### Altered Antioxidant Signaling in Manifesting Carriers of the G2019S Mutation

**Figure 2:**
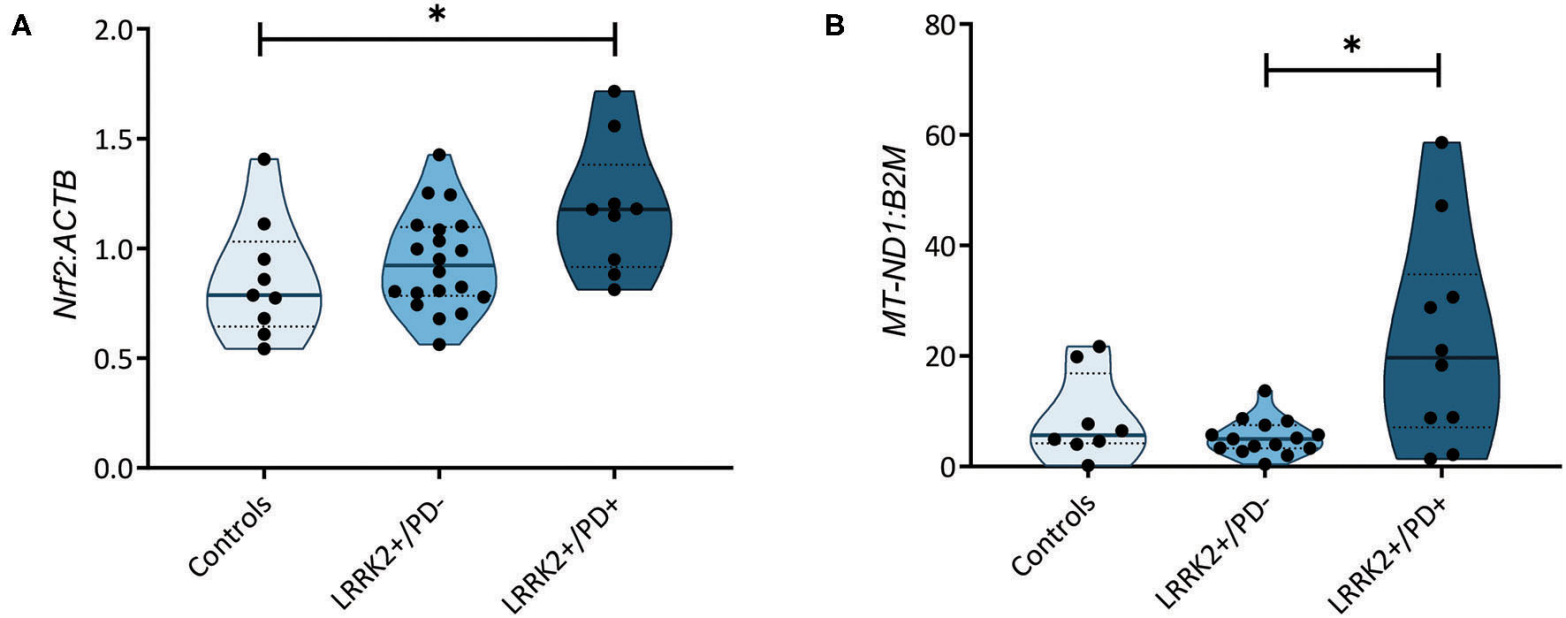
Nuclear factor erythroid 2-related factor 2 *(Nrf2)*-mediated antioxidant signaling and mitochondrial DNA (mtDNA) copy number. **(A)** *Nrf2* gene expression normalized to β-actin (*ACTB*) in controls (*n* = 9) as well as non-manifesting [leucine-rich repeat kinase-2 (LRRK2)+/PD−, *n* = 20] and manifesting carriers (LRRK2+/PD+, *n* = 10) of the G2019S mutation in LRRK2. Kruskal–Wallis test and Dunn's *post-hoc* test: controls vs. LRRK2+/PD−, *p* > 0.99; controls vs. LRRK2+/PD+, *p* = 0.03; LRRK2+/PD− vs. LRRK2+/PD+, *p* = 0.12. **(B)** MtDNA copy number in control (*n* = 8), LRRK2+/PD− (*n* = 19), and LRRK2+/PD+ (*n* = 10) individuals. Kruskal–Wallis test and Dunn's *post-hoc* test: controls vs. LRRK2+/PD−, *p* > 0.99; controls vs. LRRK2+/PD+, *p* = 0.36; LRRK2+/PD− vs. LRRK2+/PD+, *p* = 0.02. Experiments performed on three independent replicates. *p < 0.05.

A recent study measuring serum levels of the antioxidant urate in carriers of LRRK2 mutations reported reduced levels in the LRRK2+/PD+ group (15). Bakshi et al. (15) speculated that the resulting increase in ROS levels may induce the NF-E2-related factor 2–antioxidant responsive element (Nrf2–ARE) pathway. Considering this study and literature indicating that ROS induces nicks and subsequent somatic mutations in the mitochondrial genome (24), we decided to test whether our previously observed penetrance-associated mtDNA deletion phenotype (18) could be due to impaired antioxidant signaling. Quantifying the expression of the transcription factor *Nrf2* in our cohort, we observed significantly increased mRNA levels in LRRK2+/PD+ individuals (median: 1.18, IQR: 0.92–1.38) compared to controls (median: 0.79, IQR: 0.65–1.03; Kruskal–Wallis followed by Dunn's tests: *p* = 0.033). By contrast, LRRK2+/PD− individuals (median: 0.92, IQR: 0.78–1.10) showed no upregulation (Figure 2A, Supplementary Table 3).

### Increased Mitochondrial Mass in Manifesting Carriers of the G2019S Mutation

In a groundbreaking penetrance study, Fraser et al. (13) observed enhanced LRRK2 autophosphorylation in urinary exosomes from LRRK2+/PD+ compared to LRRK2+/PD− cases. In light of an established mechanistic link between LRRK2 kinase activity and lysosomal dysfunction (25), we speculated that mtDNA disintegration and elevated oxidative stress might be the consequence of impaired mitochondrial clearance in LRRK2+/PD+ individuals. To test this hypothesis, we determined the citrate synthase activity to cell number ratios as an indicator of mitochondrial mass in our fibroblast samples. We observed a significant increase (Mann–Whitney test: *p* = 0.001) in the LRRK2+/PD+ group (median: 1.74, IQR: 1.38–3.30) compared to the LRRK2+/PD− group (median: 0.95, IQR: 0.88–1.31) (Figure 1C). Moreover, mtDNA copy number analysis also revealed increased levels in LRRK2+/PD+ (median: 19.68, IQR: 7.11–34.77) compared to LRRK2+/PD− fibroblasts (median: 4.98, IQR: 3.32–7.46; Kruskal–Wallis followed by Dunn's tests: *p* = 0.024) (Figure 2B, Supplementary Table 3).

### Impaired Mitochondrial DNA Transcription Initiation in Carriers of the G2019S Mutation Independent of Disease Status

**Figure 3:**
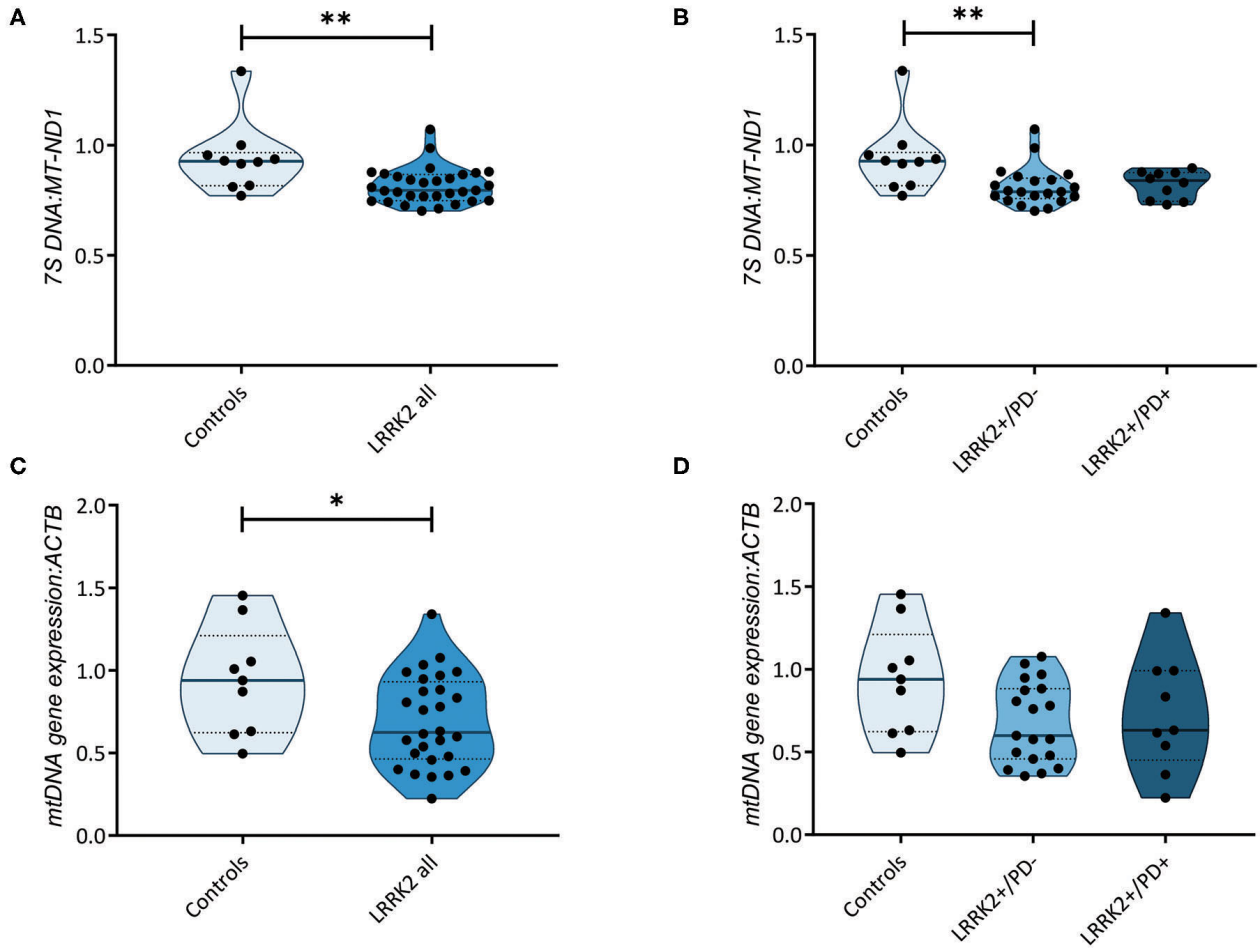
Transcription of the mitochondrial genome. **(A)** Mitochondrial DNA (mtDNA) transcription-associated 7S DNA normalized to *MT-ND1* in controls (*n* = 10) and leucine-rich repeat kinase-2 (LRRK2) G2019S mutation carriers (*n* = 31). Mann–Whitney test: *p* = 0.003. **(B)** 7S DNA*:MT-ND1* ratios in controls (*n* = 10) and non-manifesting (LRRK2+/PD−, *n* = 21) and manifesting carriers (LRRK2+/PD+, *n* = 10) of the G2019S mutation in LRRK2. Kruskal–Wallis test and Dunn's *post-hoc* test: controls vs. LRRK2+/PD−, *p* = 0.009; controls vs. LRRK2+/PD+, *p* = 0.14; LRRK2+/PD− vs. LRRK2+/PD+, *p* > 0.99. **(C)** Mitochondrial gene expression derived from averaging the mRNA levels of *MT-ND1, MT-ND4, MT-CO1*, and *MT-CYTB* in controls (*n* = 9) and LRRK2 G2019S mutation carriers (*n* = 28). Mann–Whitney test: *p* = 0.04. **(D)** MtDNA gene expression in control (*n* = 9), LRRK2+/PD− (*n* = 19), and LRRK2+/PD+ individuals (*n* = 9). Kruskal–Wallis test and Dunn's *post-hoc* test: controls vs. LRRK2+/PD−, *p* = 0.12; controls vs. LRRK2+/PD+, *p* = 0.49; LRRK2+/PD− vs. LRRK2+/PD+, *p* > 0.99. Experiments were performed using three independent replicates. **p < 0.01; *p < 0.05.

Further highlighting the role of mtDNA maintenance in determining LRRK2-PD penetrance, an increase in mtDNA transcription-associated 7S DNA was recently detected in LRRK2+/PD+ compared to LRRK2+/PD− fibroblasts (16). By measuring the abundance of 7S DNA per mtDNA molecule (*MT-ND1*), we observed a significant decrease (Mann–Whitney test: *p* = 0.003) in all LRRK2 G2019S carriers (median: 0.80, IQR: 0.75–0.87) compared to controls (median: 0.93, IQR: 0.82–0.97) (Figure 3A). However, contrary to the abovementioned study, we detected no difference in the 7S DNA:*MT-ND1* ratios between manifesting (median: 0.84, IQR: 0.74–0.87) and non-manifesting (median: 0.79, IQR: 0.76–0.85; Kruskal–Wallis followed by Dunn's tests: *p* > 0.99) carriers (Figure 3B, Supplementary Table 3).

We then tested whether mtDNA-encoded genes were differentially expressed in the LRRK2+/PD+ and LRRK2+/PD− groups. We averaged the expression of the polycistronic transcripts of *MT-ND1, MT-ND4, MT-CYTB*, and *MT-CO1*. In line with our results for the 7S DNA:*MT-ND1* ratios, we observed a significant decrease in mtDNA gene expression in all G2019S mutation carriers, independent of the disease status (median: 0.62, IQR: 0.46–0.93) compared to controls (median: 0.94, IQR: 0.62–1.21; Mann–Whitney test: *p* = 0.044) (Figures 3C,D, Supplementary Table 3).

## Discussion

There is a myriad of biological pathways implicated in LRRK2-dependent neurodegeneration including cytoskeletal dynamics, autophagy, trafficking, and mitochondrial dysfunction (1). However, the pathophysiology for penetrance of mutations in LRRK2 still remains an enigma.

Elevated phosphorylation of Ser-1292 in urinary exosomes has been shown to predict LRRK2 mutation status and risk for PD (13). Additionally, LRRK2 kinase activity has an impact on mtDNA integrity, where the LRRK2 G2019S mutants present with increased mtDNA lesions compared to kinase-dead (D1994A) mutants in rat cortical neurons (12). These mtDNA findings in model organisms seem to be translatable to patient-derived cell lines. Our group recently reported an accumulation of mtDNA deletions in LRRK2 G2019S patient-derived fibroblasts. The mtDNA deletions correlated with disease status: the manifesting carriers presented a higher load of mtDNA deletions than non-manifesting carriers and controls (18).

In this study, we further investigated mitochondrial function and related factors. In LRRK2+/PD+ compared to LRRK2+/PD− fibroblasts, we found reduced complex I activity but no changes in complex IV activity. NADH dehydrogenase deficiency is a hallmark of PD pathology (10) and has been proposed to play a role in the penetrance of LRRK2 mutations (26). Our data do not exclude the dysfunction of other mitochondrial respiratory complexes. Indeed, besides complex I, the G2019S mutation in LRRK2 also compromised the function of respiratory chain complexes III and IV in previous studies (27). Furthermore, reduced cellular ATP levels and a loss in mitochondrial membrane potential were observed in fibroblasts from PD patients harboring the G2019S LRRK2 mutation (28). However, in the context of LRRK2-PD penetrance, somatic mtDNA deletion accumulation appears to primarily affect complex I function.

Somatic mtDNA major arc deletions may be a result of impaired antioxidant signaling in LRRK2+/PD+. Situated in close vicinity to the respiratory chain, the mitochondrial genome is permanently exposed to free radicals, which can cause single- and double-strand DNA breaks. If such nicks remain unrepaired, somatic mtDNA mutations arise (24). Recently, a blood screen in ~1,500 *LRRK2*+/PD− and *LRRK2*+/PD+ individuals revealed reduced urate serum concentrations in the latter group (15). Urate can modulate antioxidant signaling, including the Nrf2–ARE pathway (15). When quantifying *Nrf2* gene expression in our samples, we found increased mRNA levels in LRRK2+/PD+ compared to control fibroblasts, suggesting a compensatory upregulation in affected individuals.

*Via* nuclear respiratory factor 1 (NRF-1), Nrf2 can act on the mitochondrial transcription factor A (TFAM), thereby interfering with mtDNA gene expression (29). During the initiation phase of mtDNA transcription, a small DNA fragment is incorporated in the D-loop region of the mitochondrial genome. This, so-called 7S DNA, can serve as a marker of mtDNA molecules undergoing transcription. A study quantifying 7S DNA in fibroblasts from four LRRK2+/PD+ and five LRRK2+/PD− cases showed elevated 7S DNA levels and an increase in mtDNA heavy-strand transcription in manifesting carriers (16). Moreover, contrary to what was previously observed in postmortem nigral neurons from idiopathic PD patients (30), the authors found increased 7S DNA:mtDNA ratios in sporadic patients compared to controls (16). When testing the abundance of 7S DNA in a larger number of controls (*n* = 10) and LRRK2+/PD− (*n* = 21) and LRRK2+/PD+ (*n* = 10) cases, we did not observe a penetrance-specific phenotype. By contrast, 7S DNA:mtDNA ratios were reduced in all individuals with LRRK2 G2019S independent of affection status. In line with these results, we detected a reduction in mtDNA gene expression in all mutation carriers. Thus, whether impaired mtDNA gene expression contributes to penetrance-associated mitochondrial dysfunction in LRRK2-associated PD warrants further studies in replication cohorts.

Overall, LRRK2 mutations cause mitochondrial dysfunction in multiple model systems including patient-derived cell lines. Mutant LRRK2 can interfere with mtDNA maintenance, mitochondrial dynamics, trafficking, and mitophagy (10, 11, 28, 31). LRRK2 pathogenic point mutations have been shown to impair mitophagy in patient induced pluripotent stem cell (iPSC)-derived neurons in a kinase-dependent manner. Wild-type LRRK2 forms a complex with Miro1, which connects mitochondria with kinesin motor proteins that transport cargo along microtubules. During the initial phase of mitophagy, LRRK2 mediates the removal of Miro1 from depolarized mitochondria, thereby reducing mitochondrial motility. In the presence of the G2019S mutation, the LRRK2-Miro1 complex is disrupted, causing delays in the induction of mitophagy (9). In light of these findings, mtDNA disintegration, respiratory chain dysfunction, and increased mitochondrial mass and mtDNA copy numbers in LRRK2+/PD+ cases may be different signs of impaired turnover of damaged mitochondria.

In conclusion, we showed that mitochondrial phenotypes such as somatic mtDNA deletions or respiratory chain complex I activity can serve as markers of LRRK2 G2019S penetrance in peripheral tissues. Further experiments are required to understand whether, unlike LRRK2+/PD− individuals, LRRK2+/PD+ cases present a faulty mitophagy system. Moreover, despite the recognized value of patient fibroblasts for PD research, inherent model limitations warrant further investigations in iPSC-derived neurons. The latter model will allow the investigation of the contribution of mtDNA disintegration to the selective death of dopaminergic neurons.

## Data Availability Statement

The datasets generated for this study are provided in the Supplementary Material. Raw data files are available on request to the corresponding author.

## Ethics Statement

The studies involving human participants were reviewed and approved by the Ethics Board of the University of Lübeck, Germany and the Luxembourg Comité National d'Éthique de Recherche (CNER). The patients/participants provided their written informed consent to participate in this study.

## Author Contributions

SD, JG, NO, LG, and CD collected the data. SD, JG, NO, and SP performed the analysis. SD, JT, SP, and AG wrote the manuscript, which was reviewed by all authors. KW, JT, JA, CK, SP, and AG conceived the study. PS, CK, and AG acquired funding for the study. AG was in charge of direction and planning of the study. All authors contributed to the article and approved the submitted version.

## Conflict of Interest

The authors declare that the research was conducted in the absence of any commercial or financial relationships that could be construed as a potential conflict of interest.
