# Supplementary material for: Mitochondrial Mechanisms of LRRK2 G2019S Penetrance
Source: Front Neurol. 2020 Aug 25;11:881. doi: 10.3389/fneur.2020.00881 (PMC7477385; doi:10.3389/fneur.2020.00881)
Supplement: Supplementary file 1 [file Data_Sheet_1.DOCX]

Supplementary Material

# Supplementary Tables

**Supplementary Table 1.** Primers and Probes used for mtDNA Status and Copy Number Analyses

| **Target** | **Forward primer** | **Reverse primer** | **Probe** |
| --- | --- | --- | --- |
| *B2M* | 5'-GCTGGGTAGCTCTAAACAATGTATTCA-3' | 5'-CCATGTACTAACAAATGTCTAAAATGGT-3' | FAM-5' -CAGCAGCCTATTCTGCCA-3'-MGB |
| *MT-ND1* | 5'-CCCTAAAACCCGCCACATCTAC-3' | 5'-GAGCGATGGTGAGAGCTAAGGT-3' | VIC-5′-CCATCACCCTCTACATCACCGCCC-3′ -TAMRA |
| *7S DNA* | 5′-CCCACACGTTCCCCTTAAATAA-3′ | 5′-CGTGAGTGGTTAATAGGGTGATAGAC-3′ | ALEXA647-5′-ACATCACGATGGATCAC-3′ -MGB |

**Supplementary Table 2.** Primers and Probes used for Gene Expression Analyses

| **Gene** | **Forward primer** | **Reverse primer** |
| --- | --- | --- |
| *Nrf2* | 5’-TTCTCCCAATTCAGCCAGCC-3’ | 5’-ACGTAGCCGAAGAAACCTCAT-3’ |
| *MT-ND1* | 5’-GAGCAGTAGCCCAAACAATCTC-3’ | 5’-GGGTCATGATGGCAGGAGTAAT-3’ |
| *MT-ND4* | 5’-ACCTACTGGGAGAACTCTCTGT-3’ | 5’-GGTGAGTGAGCCCCATTGTGTT-3’ |
| *MT-CO1* | 5’-GGAGCAGGAACAGGTTGAACAG-3’ | 5’-GTTGTGATGAAATTGATGGC-3’ |
| *MT-CYTB* | 5’-CTGATCCTCCAAATCACCACAG-3’ | 5’-GCGCCATTGGCGTGAAGGTA-3’ |
| *ACTB* | 5’-AAGGGACTTCCTGTAACAATGCA-3’ | 5’-CTGGAACGGTGAAGGTGACA-3’ |

**Supplementary Table 3.** Individual Data Points from the Current Study and from Ouzren *et al.* 2019

|  | **Individual** | **Age** | **AAO** | **Sex** | ***MT-ND4:MT-ND1* (Ouzren et al. 2019)** | **mtDNA  gene expression** | ***7S DNA:MT-ND1*** | ***NRF2*  expression** | ***MT-ND1:B2M*** |
| --- | --- | --- | --- | --- | --- | --- | --- | --- | --- |
| **Controls** | Control_1 | 72 |  | M | 1.09 | 0.61 | 1.00 | 0.79 | 4.61 |
|  | Control_2 | 33 |  | F | 1.09 | 0.94 | 0.95 | 0.77 | 21.73 |
|  | Control_3 | 63 |  | F | 1.05 | 1.45 | 0.94 | n/a | 7.71 |
|  | Control_4 | 65 |  | M | 1.10 | 0.50 | 0.92 | 0.68 | 0.18 |
|  | Control_5 | 68 |  | F | 1.05 | 1.05 | 0.91 | 1.11 | 19.87 |
|  | Control_6 | 73 |  | F | 1.00 | 1.37 | 0.82 | 1.41 | 4.93 |
|  | Control_7 | 65 |  | M | 1.10 | 0.63 | 0.77 | 0.86 | n/a |
|  | Control_8 | 68 |  | F | 1.01 | 0.87 | 1.34 | 0.95 | 4.05 |
|  | Control_9 | 59 |  | M | 1.11 | n/a | 0.93 | 0.54 | n/a |
|  | Control_10 | 42 |  | F | 1.07 | 1.01 | 0.81 | 0.61 | 6.45 |
| **Unaffected** | LRRK2+/PD-_1 | 62 |  | F | 0.99 | 0.76 | 0.81 | 0.70 | 4.98 |
|  | LRRK2+/PD-_2 | 27 |  | F | 1.02 | 0.60 | 0.70 | 0.68 | n/a |
|  | LRRK2+/PD-_3 | 57 |  | F | 1.09 | 0.50 | 0.86 | 0.99 | 0.44 |
|  | LRRK2+/PD-_4 | 55 |  | F | 0.97 | 0.88 | 1.07 | 1.25 | n/a |
|  | LRRK2+/PD-_5 | 52 |  | M | 1.00 | n/a | 0.84 | n/a | 13.70 |
|  | LRRK2+/PD-_6 | 49 |  | M | 1.04 | 0.36 | 0.87 | 0.95 | 5.72 |
|  | LRRK2+/PD-_7 | 64 |  | M | 1.01 | 0.40 | 0.77 | 0.89 | 21.32 |
|  | LRRK2+/PD-_8 | 55 |  | M | 1.01 | 0.97 | 0.74 | 1.10 | 7.46 |
|  | LRRK2+/PD-_9 | 57 |  | F | 0.98 | 0.48 | 0.71 | 1.00 | 3.32 |
|  | LRRK2+/PD-_10 | 26 |  | F | 0.97 | 0.37 | 0.79 | 0.74 | 3.70 |
|  | LRRK2+/PD-_11 | 54 |  | F | 0.98 | 0.46 | 0.78 | 1.11 | 8.20 |
|  | LRRK2+/PD-_12 | 89 |  | F | 1.04 | 0.78 | 0.99 | 1.03 | 8.67 |
|  | LRRK2+/PD-_13 | 53 |  | F | 0.99 | 1.08 | 0.84 | 0.78 | 20.82 |
|  | LRRK2+/PD-_14 | 83 |  | M | 0.95 | 0.39 | 0.82 | 0.80 | 5.19 |
|  | LRRK2+/PD-_15 | 67 |  | F | 0.98 | 0.58 | 0.88 | 0.80 | 24.18 |
|  | LRRK2+/PD-_16 | 79 |  | F | 0.98 | 0.81 | 0.77 | 1.09 | 24.88 |
|  | LRRK2+/PD-_17 | 74 |  | F | 0.99 | n/a | 0.79 | 1.43 | 2.76 |
|  | LRRK2+/PD-_18 | 54 |  | M | 0.98 | 0.95 | 0.79 | 0.56 | 5.72 |
|  | LRRK2+/PD-_19 | 55 |  | M | 0.95 | 1.03 | 0.77 | 1.24 | 3.97 |
|  | LRRK2+/PD-_20 | 51 |  | F | 1.15 | 0.87 | 0.72 | 0.81 | 3.34 |
|  | LRRK2+/PD-_21 | 66 |  | F | 1.02 | 0.58 | 0.75 | 0.82 | 2.01 |
| **Affected** | LRRK2+/PD+_1 | 66 | 65 | M | 0.99 | 0.22 | 0.79 | 0.95 | 1.41 |
|  | LRRK2+/PD+_2 | 55 | 53 | M | 0.90 | 0.62 | 0.87 | 1.15 | 58.58 |
|  | LRRK2+/PD+_3 | 85 | n/a | M | 0.95 | 1.34 | 0.73 | 1.72 | 28.78 |
|  | LRRK2+/PD+_4 | 58 | 57 | F | 1.07 | 0.83 | 0.87 | 1.18 | 8.75 |
|  | LRRK2+/PD+_5 | 59 | 43 | F | 0.92 | 0.99 | 0.88 | 1.18 | 21.03 |
|  | LRRK2+/PD+_6 | 66 | 59 | F | 0.96 | 0.63 | 0.74 | 0.88 | 30.63 |
|  | LRRK2+/PD+_7 | 79 | 53 | F | 0.93 | 0.54 | 0.89 | 1.56 | 47.19 |
|  | LRRK2+/PD+_8 | 77 | 70 | F | 0.94 | n/a | 0.83 | 2.44 | 8.90 |
|  | LRRK2+/PD+_9 | 44 | 40 | F | 0.98 | 0.99 | 0.75 | 1.20 | 18.33 |
|  | LRRK2+/PD+_10 | 71 | 63 | M | 0.97 | 0.36 | 0.85 | 0.81 | 2.18 |
